# Nanomechanics and ultrastructure of the internal mammary artery adventitia in patients with low and high pulse wave velocity

**DOI:** 10.1016/j.actbio.2018.04.036

**Published:** 2018-06

**Authors:** Zhuo Chang, Paolo Paoletti, Steve D. Barrett, Ya Hua Chim, Eva Caamaño-Gutiérrez, Maria Lyck Hansen, Hans Christian Beck, Lars Melholt Rasmussen, Riaz Akhtar

**Affiliations:** a Department of Mechanical, Materials and Aerospace Engineering, School of Engineering, University of Liverpool, L69 3GH, UK; b Department of Physics, University of Liverpool, Liverpool L69 7ZE, UK; c Computational Biology Facility, Institute of Integrative Biology, University of Liverpool, Liverpool L69 7ZB, UK; d Department of Clinical Biochemistry and Pharmacology, Center for Individualized Medicine in Arterial Diseases, Odense University Hospital, University of Southern Denmark, Denmark

**Keywords:** Arterial stiffening, Artery, Nanomechanics, Collagen, Atomic force microscopy, PF-QNM

## Abstract

The collagen-rich adventitia is the outermost arterial layer and plays an important biomechanical and physiological role in normal vessel function. While there has been a lot of effort to understand the role of the medial layer on arterial biomechanics, the adventitia has received less attention. In this study, we hypothesized that different ultrastructural and nanomechanical properties would be exhibited in the adventitia of the internal mammary artery (IMA) in patients with a low degree of arterial stiffening as compared to those with a high degree of arterial stiffening. Human IMA biopsies were obtained from a cohort of patients with arterial stiffening assessed via carotid-femoral PWV. Patients were grouped as low PWV (8.5 ± 0.7 ms^−1^, n = 8) and high PWV (13.4 ± 3.0 ms^−1^, n = 9). Peakforce QNM atomic force microscopy (AFM) was used to determine the nanomechanical and morphological properties of the IMA. The nano-scale elastic modulus was found to correlate with PWV. We show for the first time that nano-scale alterations in adventitial collagen fibrils in the IMA are evident in patients with high PWV, even though the IMA is not involved in the carotid-femoral pathway. Our approach provides new insight into systemic structure-property changes in the vasculature, and also provides a method of characterizing small biopsy samples to predict the development of arterial stiffening.

**Statement of Significance:**

Arterial stiffening occurs as part of the natural aging process and is strongly linked to cardiovascular risk. Although arterial stiffening is routinely measured *in vivo*, little is known about how localised changes in artery structure and biomechanics contributes to *in vivo* arterial stiffening. This study focusses on the role of the outermost layer of arteries, the adventitia, in arterial stiffening. The study provides data on nano-scale changes in collagen fibril structure and mechanical properties in the adventitia and shows how it relates to *in vivo* stiffness measurements in the vascular system. This is the first study to link *in vivo* arterial stiffening with nanomechanical changes in artery biopsy samples. Hence, this approach could be used to develop new diagnostic methods for vascular disease.

## Graphical abstract

**Figure:**
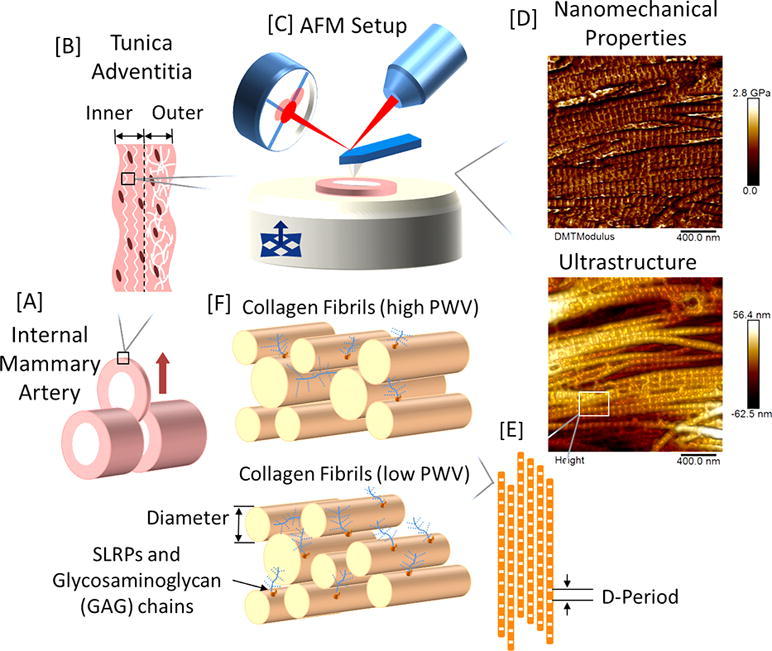

## 1 Introduction

Arteries are composite structures composed of three distinct layers, an inner intimal layer composed of endothelial cells, an elastin-rich medial layer and a collagen-rich external adventitial layer. Arteries stiffen as part of the natural aging process and *in vivo* assessment of the arterial stiffening is important for clinical diagnosis. The most commonly used technique is pulse wave velocity (PWV). PWV is based on recording the transit time of blood across two points in the vascular system and is considered a reliable method to determine arterial stiffness in routine clinical assessment [1]. Although PWV is a powerful predictor of risk of morbidity and mortality in a general population [2], it does not capture the intricate and complex structural and biomechanical processes that occur in the aging artery. Stiffness measurements derived from PWV assume that arteries are homogenous conduits, due to the inherent assumptions in the Moens-Korteweg equation on which the concept of PWV is based, although they are highly heterogeneous [3].

Arterial stiffening is associated with distinct changes across the individual layers. For example, age-related arterial stiffening is largely attributed to changes in the intima due to atherosclerosis, and degeneration of the media [4]. Furthermore, within these layers, alterations can be localized to individual components at the nano- and micro- scale [5], [6]. Hence, to understand the mechanisms driving arterial stiffening, the nano-structure and mechanical properties of individual layers within the artery need to be considered.

Most studies which are concerned with vascular pathology or aging have focused on the intima and medial layers of the artery whilst the adventitia has received less attention [7]. The medial layer has an important biomechanical role because during circumferential tension it bears approximately 60% of the load [8]. However, the mechanical role of the adventitia in normal arterial function cannot be ignored. The adventitia is dominated by circumferentially arranged, wavy collagen fibrils [8]. Due to its high collagen content, the adventitia is the stiffest layer of the artery and is thought to bear around 75% of the load during longitudinal tension [8], [9]. It becomes the mechanically dominant layer during high pressure loading [7]. The adventitia has also been found to exhibit differing nanomechanical and viscoelastic responses in different arteries, which is related to their *in vivo* physiological environment [10]. The adventitia is not only an important structural and load-bearing layer of arteries, it also plays an important physiological role in several vascular processes including atherosclerosis [11] and pulmonary hypertension [12]. The adventitia also has properties of a stem/progenitor cell niche [13]. It has been hypothesised that the adventitia may have an important role in the aging process due to a loss of function of niche-dependent signalling [13].

Here, we have employed an atomic force microscopy (AFM) method, PeakForce Quantitative Nanomechanical Property Mapping (PFQNM) [14] to investigate nano-scale properties of the adventitia in the human internal mammary artery (IMA). PFQNM enables the co-localisation of ultrastructural and mechanical properties with a high resolution. We have previously shown that this technique allows detection of regional variations in the nanomechanical properties of collagen-rich tissue [15]. In this study, we present both nanomechanical and ultrastructural data from the adventitia in a group of patients with known low or high PWV. The IMA can be collected during coronary by-pass operations and has already been established as a suitable model artery for generalized nonatherosclerotic arterial investigations because its matrix composition and biochemistry reflect alterations that occur in both the coronary and carotid arteries [16], [17], [18]. We also relate our data to the expression of small leucine-rich proteoglycans (SLRPs) which are involved in collagen fibril formation and have recently been found to be molecular targets for arterial stiffening [19]. Our fundamental study offers new insight into how nano-scale changes in the adventitia are manifested in patients with a high degree of arterial stiffening. A number of studies have previously examined the specific contribution of the adventitia to the overall biomechanical properties of arteries [7], [20], [21]. However, to the best of our knowledge, there have been no previous studies which have studied the contribution of the adventitia in relation to high PWV in humans. Furthermore, there are still very limited studies on the mechanical properties of the adventitia at the nano-level [10].

## 2 Materials and methods

### 2.1 Clinical characterization

The left internal mammary artery (IMA) was collected from 17 patients during coronary artery bypass grafting (CABG) operations and provided by the Centre of Individualized Medicine in Arterial Diseases (CIMA) (Odense University Hospital, Odense, Denmark), as part of a project approved by the Local Ethical Committee in Region Southern Denmark (S-2010044). The IMA is the repair artery for CABG operations. This study has made use of non-utilised IMA after the surgical procedure.

Prior to CABG, patients were assessed by carotid-femoral pulse wave velocity (PWV) by using the Sphygmocor system under standardized conditions as previously described in [19]. Clinical data, including age, gender, BMI, diabetes and hypertension were recorded before the surgery. The included patients were taken from a larger cohort [19] to form two groups; low PWV (8.5 ± 0.7 ms^−1^, n = 8 patients) and high PWV (13.4 ± 3.0 ms^−1^, n = 9 patients), as summarized in Table 1. The categorization of these patients as having ‘low’ and ‘high’ PWV are based on accepted reference and normal values for carotid-femoral PWV [22].

**Table 1:** Clinical parameters for IMA biopsy donors for the low (n = 8) and high PWV groups (n = 9). Mean and standard deviation (SD) or percentages are provided for each parameter. Student’s *T*-test was conducted for statistical analysis of the data. Abbreviations: BMI, body mass index; PWV, pulse wave velocity; HDL, high-density lipoprotein; LDL, low-density lipoprotein;. NS, not significant. Additional data on smoking history is provided in the Supplementary Material, Fig. S3.

Clinical parameters	Low PWV		High PWV		*P* Value
	Mean	SD	Mean	SD	
Age, y	67.9	10.7	69.9	7	NS
BMI	26.5	4.7	28.4	4.6	NS
PWV, ms^−1^	8.5	0.7	13.4	3	<0.001
Systolic blood pressure, mm Hg	131.3	18.7	154.1	28.6	NS
Diastolic blood pressure, mm Hg	77	10.7	79.4	12.3	NS
Male, sex, %	87.5		100		NS
Diabetes, %	12.5		0		NS
Hypertension, %	50		66.7		NS
Smoking, %	62.5		88.9		NS
Total cholesterol, mmolL^−1^	4.1	0.5	4.9	1.5	NS
P-Cholesterol LDL, mmolL^−1^	2.1	0.5	3.1	1.3	NS
P-Cholesterol HDL, mmolL^−1^	1.2	0.1	1.1	0.3	NS
P-Triglyceride, mmolL^−1^	1.6	0.6	1.6	0.5	NS
P-creatinin, mmolL^−1^	89.8	19.9	91.6	20.4	NS
HbA1c (glycated haemoglobin (A1c)	0.06	0.007	0.06	0.002	NS

### 2.2 Peakforce Quantitative nanomechanical Mapping (PFQNM) Atomic force microscopy (AFM)

Immediately after surgery the IMA vessels were embedded in optimal cutting temperature (OCT) compound (Tissue-Tek Sakura Finetek, The Netherlands) and immediately frozen at −80 °C after snap freezing. The unfixed, frozen samples were then cryosectioned to a nominal thickness of 5 μm using a Leica CM1850 cryostat (Leica Microsystems (UK) Ltd, Milton Keynes) and adhered onto glass coverslips. All the cryosections were stored in a −80 °C freezer until testing. AFM imaging was conducted using the PFQNM method with a MultiMode 8 Atomic Force Microscopy (AFM) (NanoScope VIII MultiMode AFM, Bruker Nano Inc., Nano Surface Division, Santa Barbara, CA). Before measurement, the coverslips were mounted on metal stubs. The tissue sections were de-thawed at room temperature for 15 min and then washed with ultrapure water for 10 min to remove any excess OCT compound. Once the sample was fully dehydrated, the adventitia was imaged with AFM. All measurements were carried out at room temperature (21 °C) with Bruker RTESPA-150 etched silicon probes. These probes have a nominal radius of 8 nm and a cantilever with a nominal spring constant of 5 Nm^−1^ and resonant frequency of 150 kHz. The spring constant and tip radius of the probe were calibrated prior to sample measurement. In addition, a photostress coating polymer reference sample (PS1, Vishay Precision Group, Heilbronn, Germany) with known elastic modulus was used to calibrate the elastic modulus measurements [14].

**Fig. 1:**
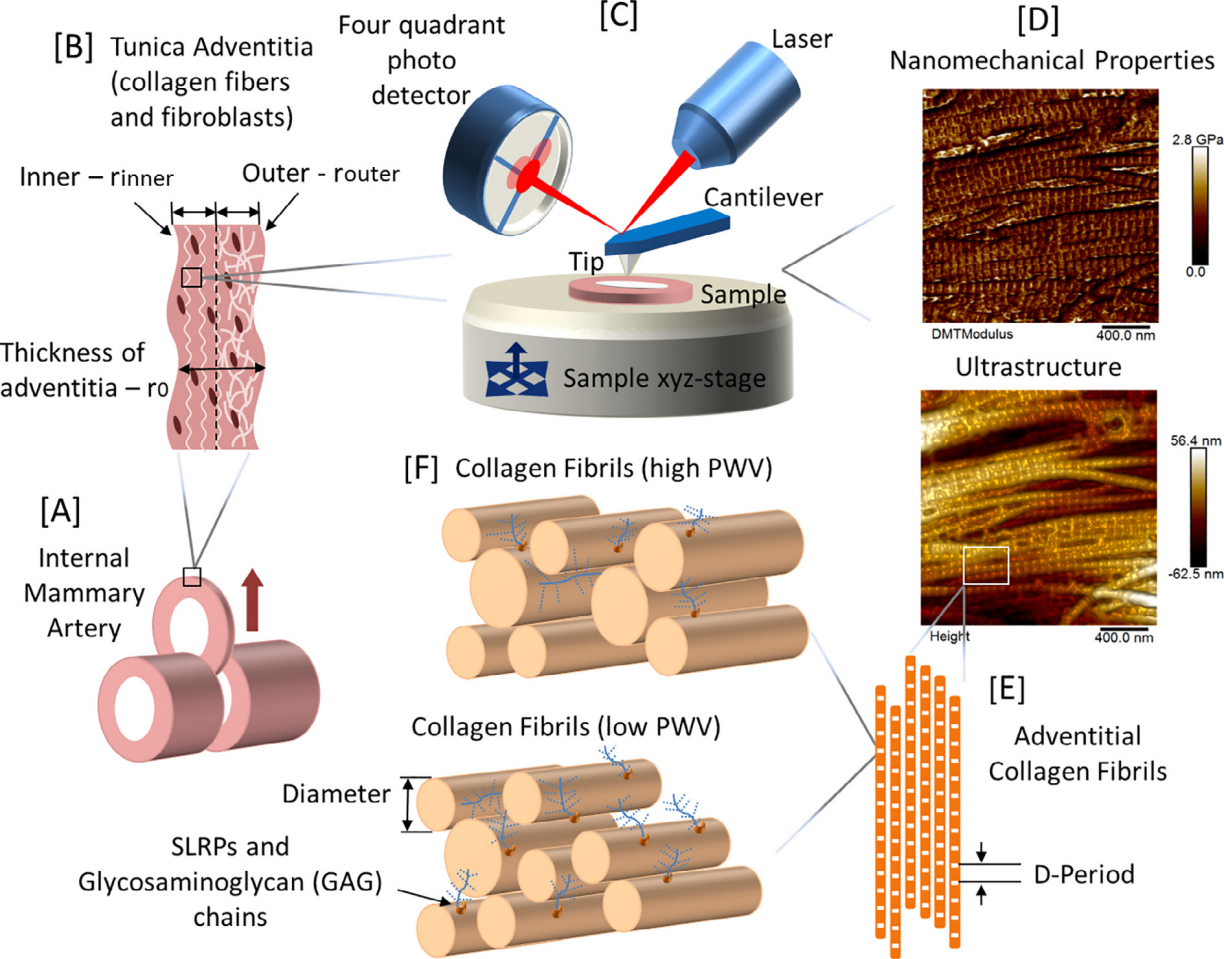
Schematic representation of the experimental approach (A) IMA sections (B) Location of adventitia for testing. Image analysis routine: (C) AFM imaging (D) Nanomechanical property measurement with PFQNM (E) and (F) Collagen fibril characterisation.

Using the optical microscope integrated with the AFM setup, the inner adventitia (Fig. 1) was identified and the probe was placed over it prior to any testing. All testing was conducted with a scan rate of 0.93 Hz, a resolution of 256 pixels per line and with two fixed scan sizes (2 × 2 μm for mechanical and collagen morphological measurements and 5 × 5 μm for topographical observation). Along with topographical images, the PFQNM method produced elastic modulus maps as described earlier. Thus, each image was composed of 65,536 measurements for mechanical property comparisons (256 × 256 independent force curves). For each patient, 2 tissue sections were imaged and 3 random images were collected from each section for analysis, resulting in 6 images per patient. This added to a total of 48 and 54 images analyzed for low and high PWV groups respectively. All of the raw AFM files were processed and analyzed to yield the mean elastic modulus for each image with NanoScope Analysis version 1.5 (Bruker, Santa Barbara, CA). The force curves were fitted using the Derjaguin-Muller-Toporov (DMT) analytical model as outlined by Young et al. [14].

### 2.3 Collagen fibril analysis

**Fig. 2:**
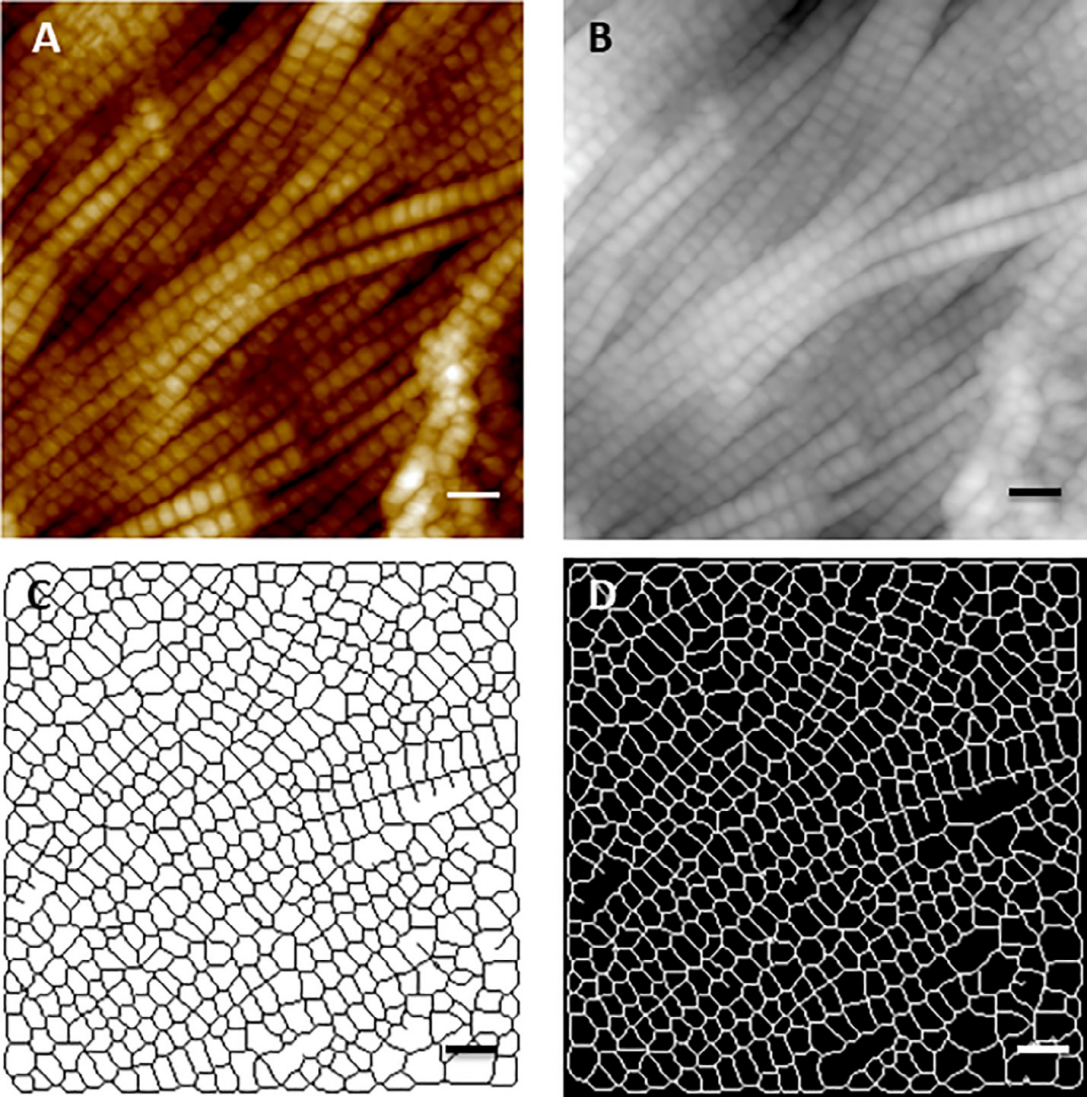
Image analysis routine: (A) Original 2 × 2 μm AFM topography image; (B) Loaded images in Image SXM; (C) Thresholded and skeletonized image; (D) Valid rectangles in the image. Scale bar represents 200 nm.

Collagen fibril diameters and D-Periods were analyzed using a custom routine for Image SXM [23]. Following image capture, the raw image (Fig. 2A) was opened with Image SXM (Fig. 2B) and a convolution filter was applied with appropriated threshold adjustment, by which the AFM image was converted to a binary black and white image. In the image, individual collagen fibril can be considered as consisting of contiguous rectangular objects in which the width and length is equivalent to the diameter and D-Period of the fibril. Subsequently, several pixels were added or removed from the edges of these rectangles in the binary image to seal or dilate these rectangles for more accurate measurement, and then pixels from the edges of these rectangles were removed until they were reduced to one pixel wide skeletons (Fig. 2C). This procedure was used to analyze over 3000 rectangles for each group (Fig. 2D), to obtain collagen fibril diameter and D-Periods. In total, 102 and 148 images were analyzed in this manner for 7 patients with low PWV and 9 patients with high PWV. Images from 1 patient (patient 559) were composed of loosely packed fibrils and hence were unsuitable for the image analysis routine which considered the fibrils as contiguous rectangular objects (see Fig. S1, Supplementary Material). Thus, fibril morphology measurements for this patient were discarded.

### 2.4 Histology

To ensure that the vessel did not contain atherosclerosis or other pathologies and to judge the location of the external elastic membrane and the media-adventitia border, standard histological staining was performed on frozen sections with Mason Trichrome (connective tissue stain) and Weigert’s elastin staining as previously described [18].

The intima-media thickness was assessed from the Weigert’s elastin stained images by calculating the circumference of the medial and intima rings, and then determining the difference in the radius of the two rings. These measurements were made in ImageJ [24] for all 8 and 9 patients in the low and high PWV groups respectively.

### 2.5 Integration of quantitative proteomics, nanomechanical data and patient metadata

Quantitative proteomics data of for some of the patient cohort analysed in this study were available from a larger cohort study published by Hansen et al. [19]. Full methodological data for these measurements can be found in their paper. The data available was for the 7 SLRP proteins that were found to be downregulated in the high PWV patients, namely decorin, biglycan, mimecan, lumican, prolargin, podocan and asporin [19]. With the aim of integrating the nanomechanical and structural data acquired in this project with both proteomics data and patient metadata, a multivariate transformation called principal component analysis (PCA) has been used.

### 2.6 Statistical methods

All the data are presented as mean ± SD and showed as mean ± SEM on all of the bar charts. The majority of the statistical analyses were tested using OriginPro, version 9 (OriginLab, Northampton, MA). All of the patient measurements were averaged prior to statistical analysis. Group differences were assessed via suitable 2-sample independent tests selected after appraisal of data normality and homoscedasticity. Patient clinical characteristics in the 2 groups were analyzed with the Student’s *t*-test. Mann-Whitney *U* Test was used to compare the nanomechanical properties and the morphology of adventitial collagen fibrils in low and high PWV groups. To assess for statistical significance in distributions of D-Period and diameter of adventitial collagen fibrils and nanomechanical properties between the low and high PWV groups, Kolmogorov-Smirnov (K-S) tests were applied to the data. Spearman's Rank Order Correlation was used to obtain the correlation coefficient for the elastic modulus-PWV relationship and a correlation test was used to test its significance. All statistical tests were corrected for false discovery rate via Benjamini and Hoghberg method [25]. Proteomics data were analyzed in junction with nanomechanical variables and quantitative metadata variables (i.e. age, BMI, cholesterol and HbA1c) of all the patients via the unsupervised data transformation principle component analysis (PCA). Patients with any missing values (reported in Table S1, Supplementary Material) were not included in the analysis. Data was mean centered and scaled prior to PCA analysis. The PCA analysis was performed in the statistical software R [26].

## 3 Results

### 3.1 Histological analysis

**Fig. 3:**
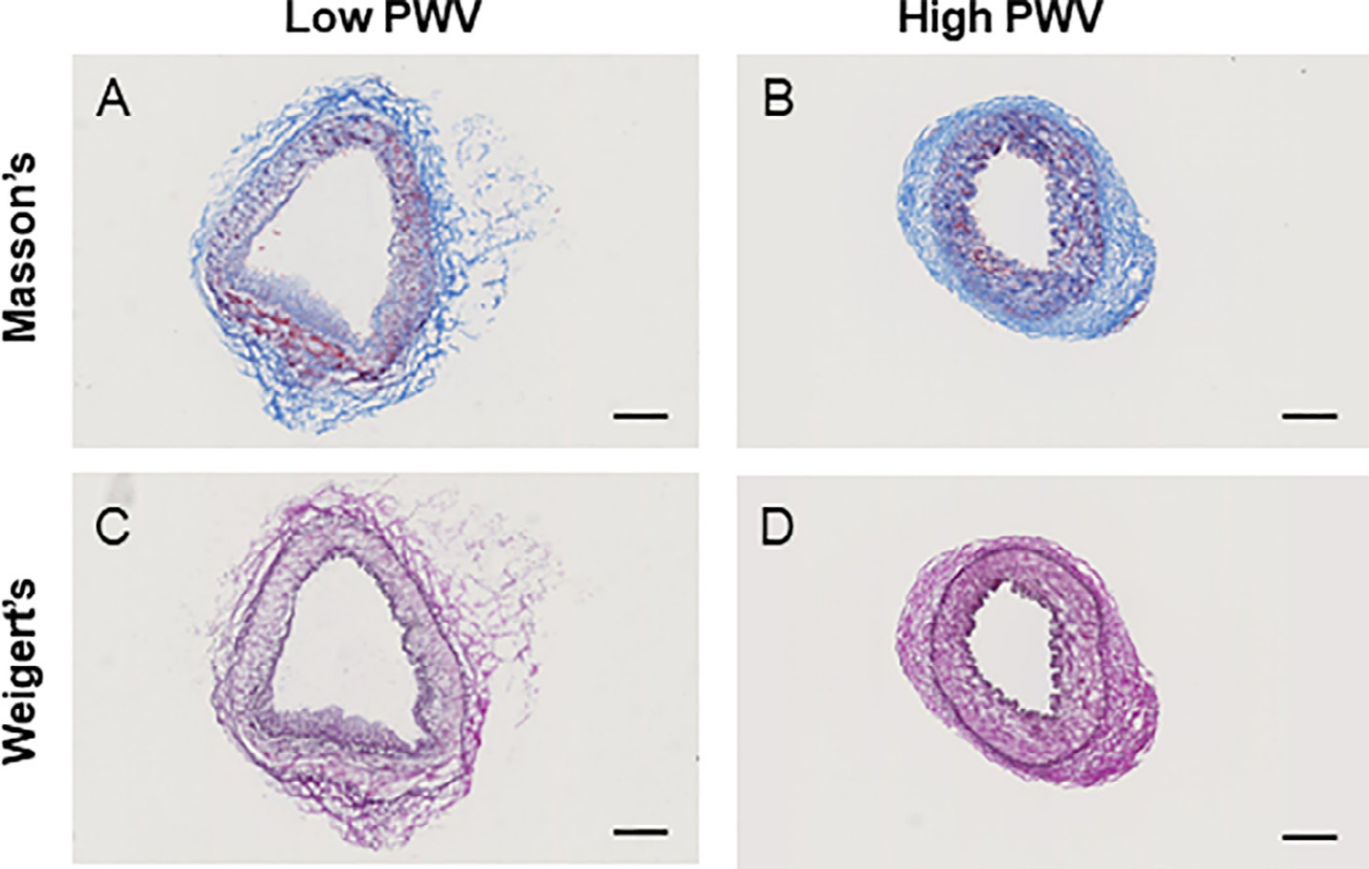
Histological sections for the IMA (A) and (B) Masson’s staining for collagen in low (A) and high PWV (B) groups (C) and (D) Wiegert’s staining for elastin in low (C) and high PWV (D) groups. Scale bar indicates 200 μm.

A histological study was undertaken on IMA vessels to assess the integrity of all tissue layers and to evaluate the presence of collagen and elastin. Representative histological sections of IMA vessels for both low and high PWV groups are shown in Fig. 3. Collagen and elastin were present in the histological sections of both groups and the border between the media and adventitia could clearly be identified classifying these sample vessels as normal. It should be noted that the IMA is a transition artery which is typically muscular with little or no elastin, but it can have high elastin content in some individuals [27]. Each of the IMA samples were characterized from the histology images and were all found to be muscular with low elastin content except for one patient in each group which had a moderate amount of elastin. Example images from these two patients (patient 559 and patient 620) are presented in the Supplementary Material (Fig. S2). As this study focusses on adventitial collagen, elastin content in the media was not consider a factor of influence in this study.

The intima-media thickness, as determined from the histology images, was 162.10 ± 78.3 μm and 209.3 ± 76.6 μm in the low and high PWV. The non-parametric Mann-Whitney *U* test demonstrated that there was no statistically significant difference between the two groups (p-value = 0.19).

### 3.2 Nanomechanical properties

We used Peakforce Quantitative Nanomechanical Mapping (PFQNM) to capture high-resolution mechanical property maps along with collagen fibril morphology in the adventitia. These nano-scale measurements were compared to the clinical assessment of ‘arterial stiffness’ via carotid-femoral PWV. A total of 17 patients were analyzed and 6 measurements were obtained for each. These were further averaged and statistical tests calculated.

**Fig. 4:**
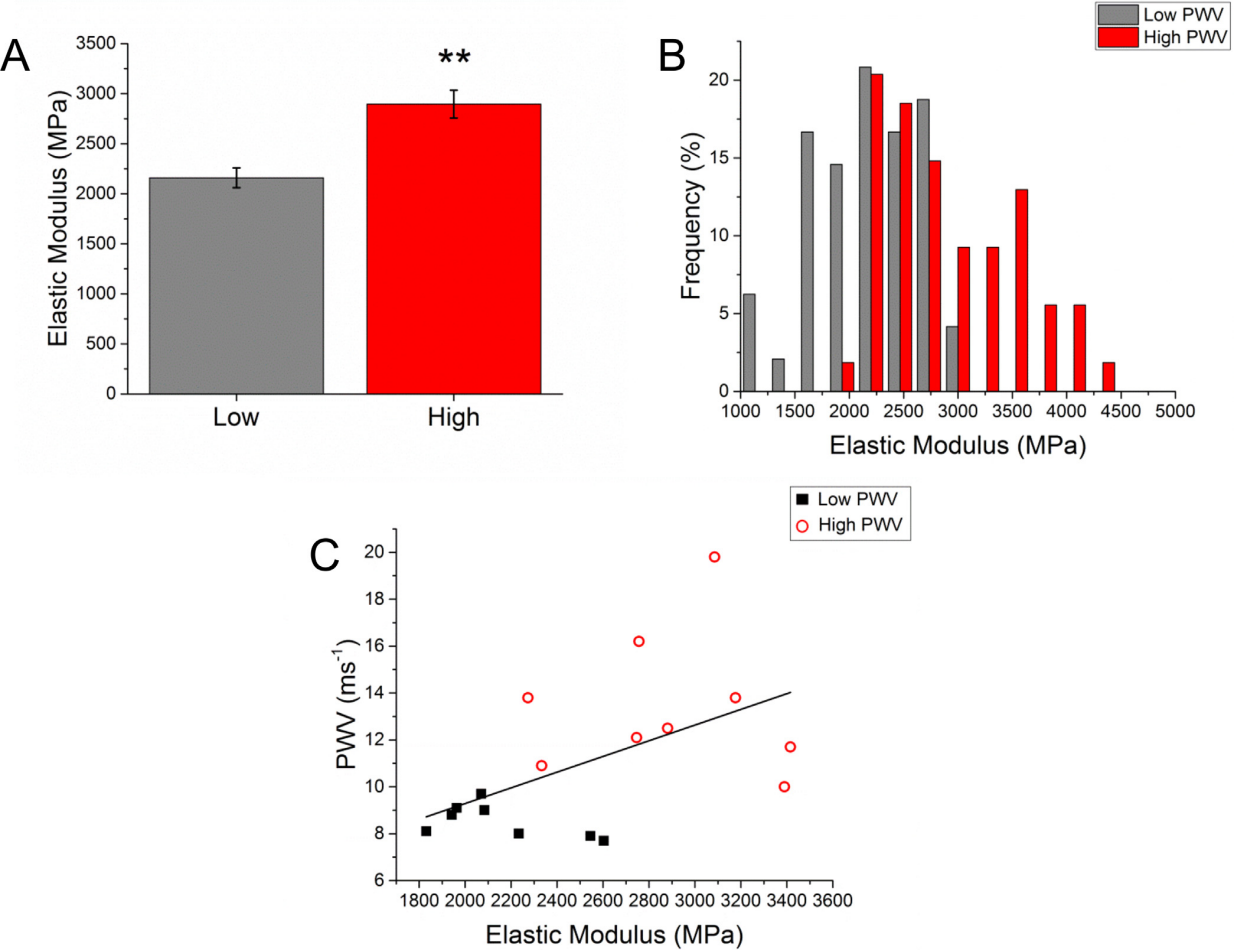
Nanomechanical properties of the adventitia for the low and high PWV group. (A) Bar graph showing mean ± SEM (n = 8 and n = 9 patients in the low and high group). Asterisks represent a significance difference between both groups with p-value < 0.01 (B) Elastic modulus distribution in each group (n = 48 and 54 measurements in the low and high PWV groups Kolmogorov-Smirnov, p < 0.001). (C) A positive correlation of 0.56 between PWV and elastic modulus was found when data from both groups was pooled together (Spearman’s Rank Order Correlation, p-value = 0.02).

The differences in elastic modulus between both groups of patients, i.e. low and high PWV (see Fig. 4A), was tested via the non-parametric Mann-Whitney *U* test that resulted in a significant test (see details in Table 2). The difference distribution of the elastic modulus measurements is shown in Fig. 4B. A Kolmogorov-Smirnov test was performed resulting in a significant difference (p-value < 0.001) in terms of elastic modulus distributions for both low and high PWV groups, indicating the significant heterogeneity at the sub-micron range for this variable. Furthermore, as shown in Fig. 4C, Spearman’s correlation between PWV and elastic modulus was calculated to be 0.56 and tested and shown to be significant (p-value = 0.02).

**Table 2:** Elastic modulus summary statistics. Mean, standard deviation (SD) and sample size (n) for each cohort are provided for the low and high PWV groups. Results from the non-parametric Mann-Whitney *U* test are summarized.

Group	Elastic modulus (MPa)			Statistical test	95% CI	p-value	Adjusted p-value
	n	Mean	SD				
Low PWV	8	2159.3	282.5	Mann-Whitney	(−1154.8, −309.8)	9.8 × 10^−4^	0.003
High PWV	9	2895.2	414.4				

### 3.3 Morphology of adventitial collagen fibrils

**Fig. 5:**
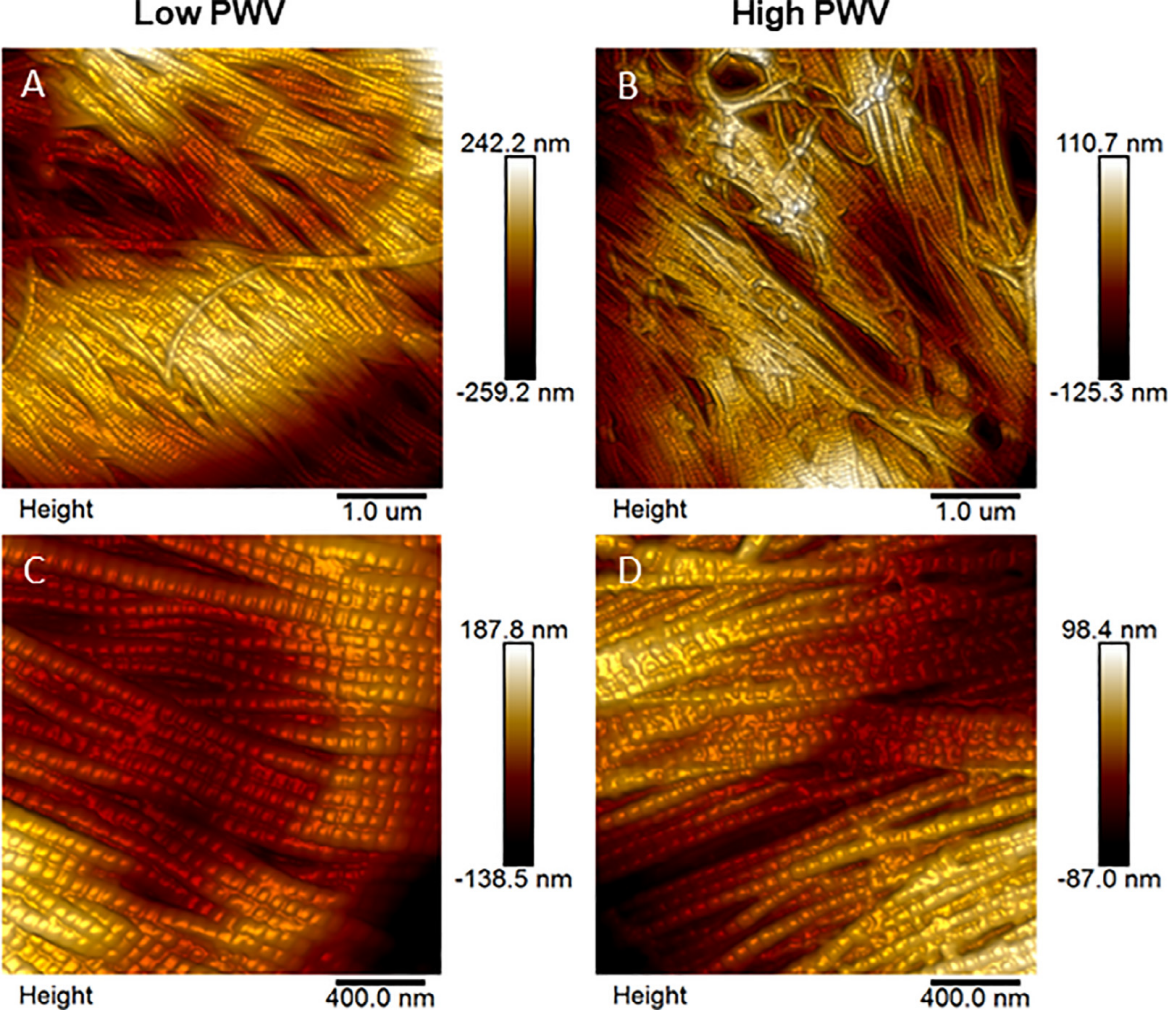
AFM topography images showing collagen fibrils in the adventitia. Example images are shown for the low (A) and high (B) PWV groups at 5 × 5 μm. Typical 2 × 2 μm images are also shown for the low (C) and high (D) PWV groups. At this scan size, individual collagen fibrils were clearly visible with their characteristic D-Period.

We subsequently compared collagen fibril morphology in the adventitia for the low and high PWV groups. Abundant collagen fibrils were observed in the adventitial layer with AFM in both groups (Fig. 5). The collagen fibrils were found to be tightly packed and highly aligned (Fig. 5A and B). In some instances, the fibrils were orientated transverse to the principal direction. In the smaller scan sizes (2 µm × 2 µm), the D-Period and diameter of individual collagen fibrils could clearly be seen (Fig. 5C and D).

For each of the patients, an average of 460 and 450 measurements were taken from the AFM images for collagen fibril diameter and D-Period respectively. All measurement data obtained for each patient were averaged.

**Fig. 6:**
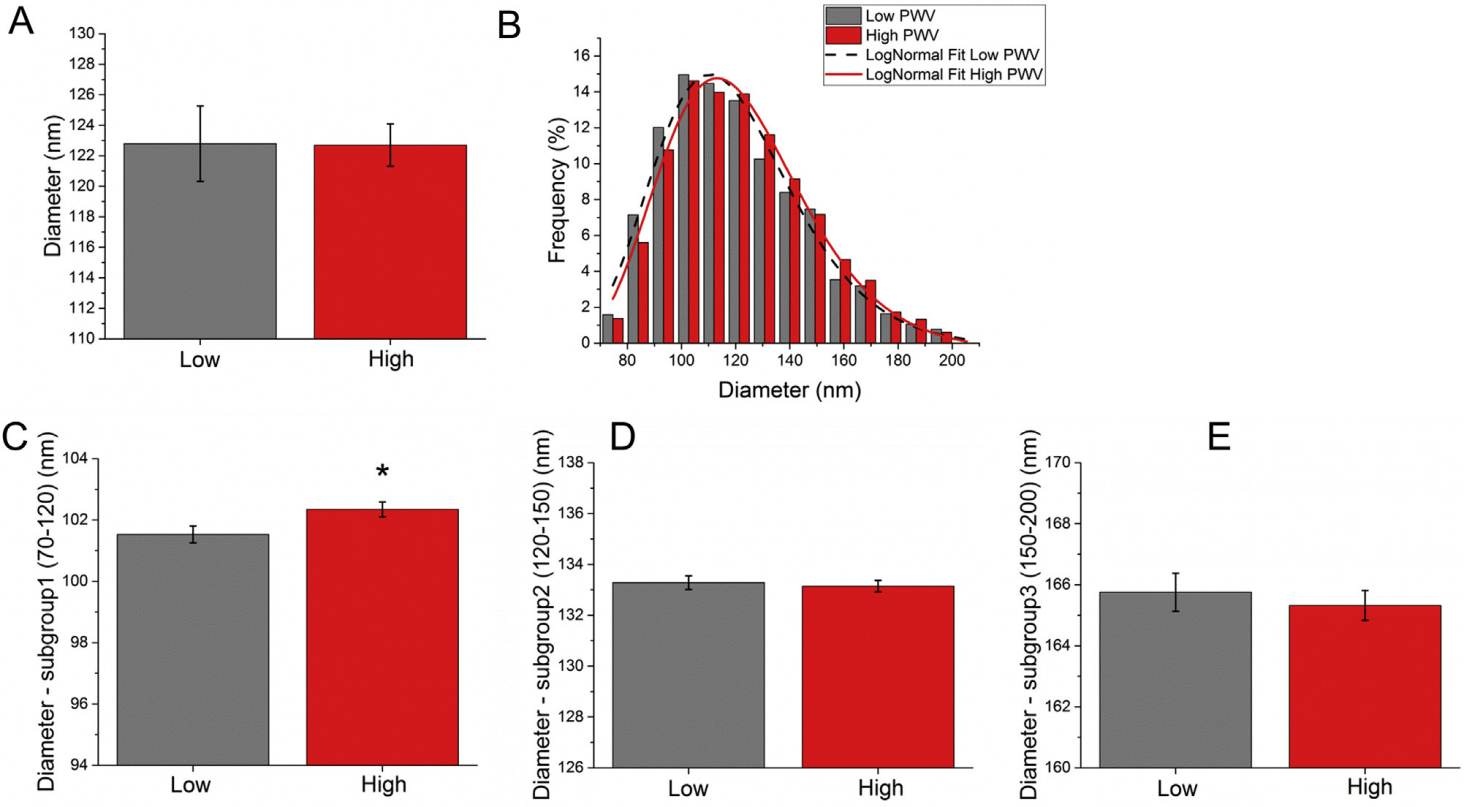
Collagen fibril diameter in the low and high PWV groups (A) Bar graph showing mean ± SEM (n = 7 and n = 9 patients in the low and high group) (B) Distribution of diameters in the two groups (n = 3228 and 4141 measurements in the low and high PWV groups respectively). The distributions were statistically different. (C)–(E) Diameter values shown in 3 sub-groups; 70–120 nm (C), 120–150 nm (D) and 150–200 nm. There was a statistically significant difference in the low sub-group, 70–120 nm (non-parametric Mann-Whitney *U* test, adj p-value = 0.049).

Collagen fibril diameters are shown in Fig. 6. Differences in collagen fibril diameter between both groups of patients, i.e. low and high PWV (see Fig. 6A), were tested via the non-parametric Mann-Whitney *U* test that resulted in a non-significant p-value (p-value = 0.8). Summary statistics are shown in Table 3. Despite not finding overall mean differences we observed that low PWV patients presented a larger number of small diameter fibres than high PWV patients e.g. 55.6% of fibrils were <120 nm in the low PWV group as compared to 51.4% in the high PWV group. Consequently the fibril size distributions were tested via a Kolmogorov-Smirnov test and a statistically significantly different was found (p-value = 0.0015). The distributions are shown in Fig. 6B. Due to these differing distributions, we further analyzed the fibril diameters by splitting the diameters into three size groups as shown in Fig. 6C–E. We determined significance with the Mann-Whitney test between both groups at these diameter interval: 70–120 nm, 120–150 nm and 150–200 nm. These subgroups were chosen as they are in agreement with previous studies focusing on collagen fibril ranges [28], [29]. The Mann-Whitney *U* test yielded a statistical significant difference for the 70–120 nm group, with the mean diameter being higher for the high PWV group (adjusted p-value = 0.049). Full details for the statistical tests can be found in the Table 3.

**Table 3:** Collagen fibril diameter and D-Period summary for the low and high PWV groups. Mean and standard deviation (SD) and sample size are provided together with the results of the Mann-Whitney test for each variable.

		n	mean	SD	95%CI	p-value	Adjusted p-value
*Collagen fibril diameter*							
All	Low	7	122.8	6.6	(−6.41, 6.81)	0.84	0.840
	High	9	122.7	4.2			
Subgroups (nm)	Percentage (%)	mean	SD				
70–120	Low	55.6	101.5	11.5	(−1.50, −0.05)	0.03	0.049
	High	51.4	102.3	11.1			
120–150	Low	30.9	133.3	8.5	(−0.55, 0.82)	0.72	0.860
	High	33.7	133.1	8.5			
150–200	Low	13.5	165.7	13	(−1.16, 1.28)	0.94	0.940
	High	14.9	165.3	12.2			

*Collagen fibril D-Period*							
		n	mean	SD	95%CI	p-value	Adjusted p-value

All	Low	7	62.4	1.4	(−1.66, 1.62)	0.76	0.840
	High	9	62.5	1.3			
Subgroups (nm)	Percentage (%)	mean	SD				
45–59 nm	Low	31.8	54.5	3.3	(0.25, 0.77)	9.31 × 10^−5^	5.58 × 10^−4^
	High	36.9	53.9	3.6			
59–70	Low	50.3	64.1	3.1	(−0.13, 0.29)	0.47	0.71
	High	43.3	64	3.1			
70–80	Low	17.9	73.9	2.7	(−0.63, −0.06)	0.01	0.046
	High	19.8	74.2	2.8			

**Fig. 7:**
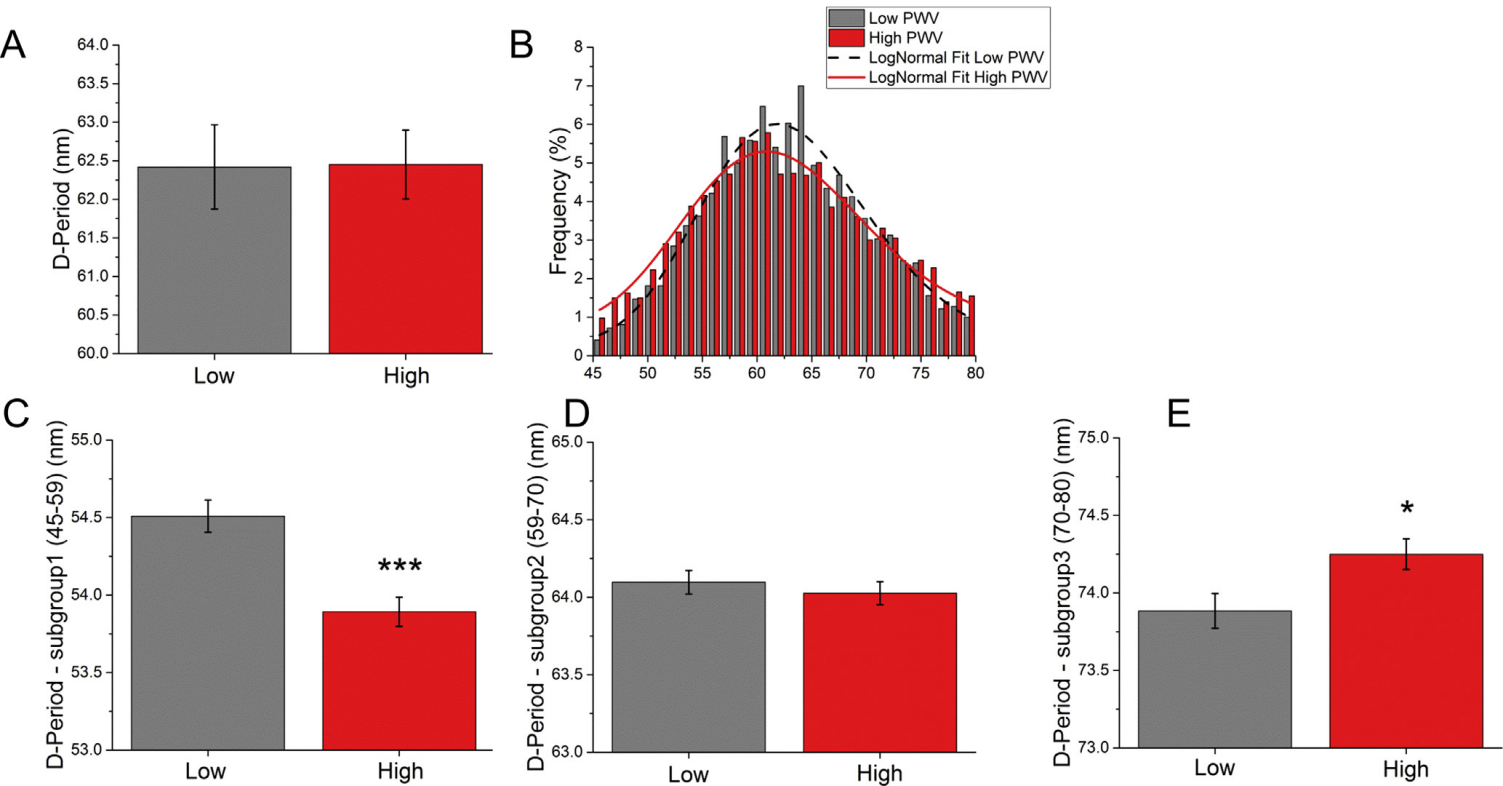
Collagen fibril D-Period in the low and high PWV groups . (A) Bar graph showing mean ± SEM (n = 7 and n = 9 patients in the low and high group) (Mann-Whitney *U* test, pvalue = 0.8) (B) Distribution of diameters in the two groups (n = 3201 and 3994 measurements in the low and high PWV groups respectively). The distributions were statistically different (Kolmogorov-Smirnov, p-value < 0.0001). (C)–(E) D-Period values shown in 3 sub-groups. There was a statistically significant difference in the low sub-group, 45–59 nm (Mann- Whitney *U* test, adj p-value < 0.0001) and also in the high sub-group, 70–80 nm (Mann- Whitney *U* test, adj p-value = 0.046).

Collagen fibril D-Period data are shown in Fig. 7 and summarized in Table 3. Similar to the diameter analysis, differences in collagen fibril D-Period between both groups of patients, i.e. low and high PWV (Fig. 7), were tested via the non-parametric Mann-Whitney *U* test that resulted in a non-significant p-value (p-value = 0.8). However, a statistically significant difference via Kolmogorov-Smirnov test was found in the D-Period distribution between the two groups (p-value < 0.0001), as shown in Fig. 7B. In the high PWV group, 36.9% fibrils had a reduced D-Period (<59 nm) as compared to 31.8% in the low PWV group. Furthermore, in the high PWV group, there were less fibrils with D-Periods in the expected range of 59–70 nm (low PWV = 50.3%; high PWV = 43.3%). The expected ranges were determined from other studies [30], [31]. These differences are further highlighted in Fig. 7C–E where the D-Period ranges are split into 3 groups; 45–59 nm, 59–70 nm and 70–80 nm. The centre group, 59–70 nm covered the ‘expected range’ whereas the two tail groups (45–59 nm and 70–80 nm) represented those D-Period values which are far from the expected values. The Mann-Whitney *U* Test revealed that there were statistically significant differences in the 45–59 nm (adjusted p-value < 0.0001) and 70–80 nm groups (adjusted p-value = 0.046).

### 3.4 Principal component analysis

**Fig. 8:**
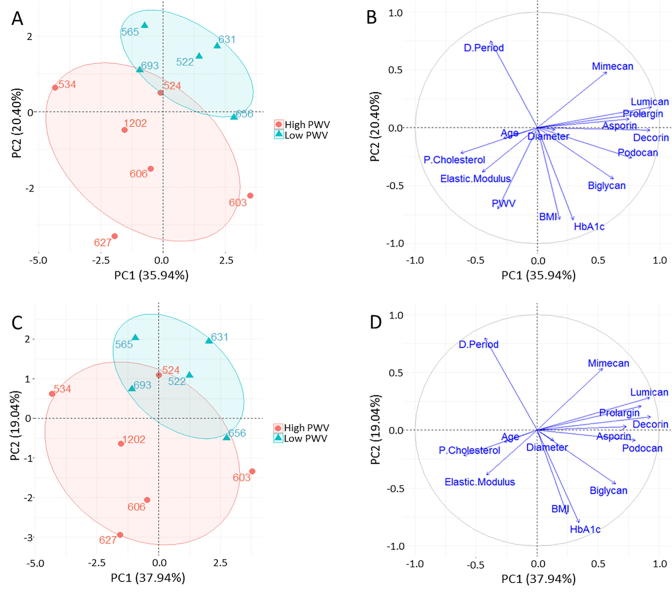
Principal component analysis (PCA) of patient proteomics, quantitative metadata and nanomechanical variables. (A) Score plot of the two first principal components. Each dot represents a patient. Patients coloured by group, ellipses represent the 67% region around the mean of the points of each group. (B) Loading plot of (A) showing the variables that contribute the most to the structured observed in (A); PWV and Elastic modulus are two of the most contributing variables to the separation between both groups. (C) Score plot of the two principal components of patient data without the PWV variable. Similar separation between both groups can be observed. (D) Loading plot of (C) shows how elastic modulus is one of the most contributing variables to the separation observed. Of the SLRPs, mimecan presents the most contribution to the separation between groups observed.

With the aim of studying the relations between the proteins published in [19] and the variables measured in this study were summarized and integrated in the multivariate transformation Principal Component Analysis (PCA) to assess data structure as described in the methods. The score plot for this transformation is shown in Fig. 8A where it can be appreciated that there is a difference between high and low PWV patients. The variables contributing the most to this separation are shown in the loading plot (Fig. 8B) where not surprisingly we can see how PWV is one of the major contributors to the separation of the patients. Interestingly and in agreement with our data it is closely correlated with the elastic modulus. Furthermore PCA on the data without the PWV variable shows similar separation between both high and low PWV patients and shows elastic modulus as one of the key variables to the separation (Fig. 8C and D). Interestingly, D-Period and collagen fibril diameter were also found to be negatively correlated. Most SLRPs were closed grouped in the PCA analysis with some differences in mimecan and biglycan. Mimecan was found to be one of the most contributing variables to the separation observed between groups. Biglycan was found closely related to collagen diameter.

## 4 Discussion

In this study, we used the IMA as a model vessel to understand how the adventitia is altered in patients with a high degree of arterial stiffening. It is a suitable artery for *in vitro* arterial stiffening studies, since it is readily accessible as the repair artery during coronary artery bypass operations and hence it was available for our study from a well-characterized group of patients. The measure of ‘arterial stiffening’ used to characterize patients in our study was carotid-femoral pulse wave velocity (PWV). Carotid-femoral PWV is an established measure of arterial stiffness *in vivo* and is determined from the time taken for the arterial pulse to propagate from the carotid to the femoral artery [32]. Although the stiffness of the IMA does not contribute to the carotid-femoral PWV measurement itself, we have already shown that it is a model vessel for arterial stiffening studies and reflects more systemic changes in the vasculature [19]. This has also been highlighted by other studies. For example, matrix metalloproteinase-2 (MMP-2) activity in the IMA has been found to be associated with age, hypertension and diabetes [33], [34]. Furthermore, Fibulin-1, an important extracellular matrix (ECM) protein involved in matrix organization, has been found to accumulate in the wall of the IMA of patients with type 2 diabetes [17]. Hence, each of these studies demonstrates that ECM changes in the IMA reflect generalized arterial alterations related to vascular stiffness, diabetes and other risk factors.

### 4.1 Nanomechanics of the adventitia

All previous studies that have utilised the IMA as a target to understand systemic changes associated with cardiovascular disease have reported global data for the IMAs e.g. with the use of destructive biochemical methods that do not discriminate across the different layers of the vessel [19] or with measurements across the intima-media layers [17]. Given that the IMA is now an established model for vascular disease, we sought to focus on the role of the IMA adventitia in arterial stiffening. Grant and Twigg [10] demonstrated that the nanomechanical and viscoelastic properties of the adventitia of the porcine aorta and pulmonary artery exhibit distinct differences which are in line with their functional properties. Our study now demonstrates that the nanomechanical properties of the IMA adventitia can help discriminate between distinct patient cohorts. Specifically, our data demonstrate that the IMA adventitia exhibits different mechanical properties at the level of individual collagen fibrils in patients with low PWV as compared to those with elevated PWV. The changes are manifested as localised stiffness increases in the adventitia in high PWV patients, as determined with AFM, and by ultrastructural changes (see 4.2, 4.3). We have previously demonstrated that downregulation of certain SLRPs in the IMA occurs in patients with high PWV [19]. Downregulation of SLRPs is particularly relevant for the adventitia because SLRPs are involved in collagen fibril formation [35], [36]. Collagen fibril morphology is discussed further in the following sections.

### 4.2 Collagen fibril diameter

Our study focused on the inner adventitia and the resulting fibril diameters are in agreement with the work of Merrilees et al. [28] in which they report the mean-fibril diameter ranges from 50 to 100 nm in the adventitia of arteries from human, pig and rat. Merrilees et al. observed that artery collagen fibril diameters were inversely correlated with glycosaminoglycan (GAG) levels. Furthermore, they suggested that fibril diameters serve as excellent markers for determining glycosaminoglycan (GAG) levels in samples which are too small to sample biochemically. Fibril/proteoglycan interactions are responsible for stress transfer within the ECM and are altered with changes in fibril diameters [37]. Our PCA analysis hints at a complex interaction between collagen diameter and SLRPs. Biglycan and collagen diameter are relatively correlated but they have a different direction in space with respect to lumican, prolargin, decorin, asporin and podocan. The important role of biglycan and decorin in governing collagen fibril structure has been highlighted in a study on a compound knockout model of mouse tendon (i.e. both biglycan and decorin) where there was a shift to larger diameter fibrils and also a shift in the diameter distribution [38].

The main difference in fibril diameter between low and high PWV groups that we observed was a different distribution of the collagen fibril diameters with high PWV patients showing less fibrils with small diameters (70–120 nm). Although there have been previous studies that have reported collagen fibril diameters in arteries [28], [39], little is known about how collagen fibril diameters change in vascular tissue with aging or with vascular stiffening. Our most developed understanding of the role of collagen fibril diameter in tissue biomechanics arises from studies on rat tail tendon. An important study in this area is the work by Goh et al. [37]. They developed a strain energy modelling approach to understand how collagen fibril diameter contributes to tendon resilience and resistance to failure. They suggested that their findings may have broader applicability to other connective tissues including vascular tissues. The Goh et al. model demonstrates that fibril size is related to strain energy density and that failure of the ECM is more likely to occur with smaller fibril diameters. This is because fracture is easier due to lower strain energy being absorbed; an increase in large diameter fibrils with a decrease in smaller diameter fibrils contributes to an increase in strain energy density. This is the trend we found in the high PWV group. Hence, we speculate that this change in fibril distribution, especially in small diameter collagen fibrils (<120 nm), serves as a protective mechanism i.e. this provides resilience and resistance to the vessel as a response to wider pulse pressure and higher cardiac load associated with arterial stiffening.

The limitations of the Goh et al. model for tendon work are that the rat tail tendon is not weight-bearing and may be influenced by systemic effects of aging [37]. However, these are not limitations when applied to the IMA due to the reasons stated earlier; the IMA does not contribute to carotid-femoral PWV, and it is also affected by systemic effects in the vasculature.

### 4.3 Collagen fibril D-Period

Collagen fibril D-Period is typically reported during *in situ* loading for soft tissues e.g. [40] where the D-Period provides an indication of collagen fibril strain during loading. Very small changes in D-Period provide an important indication of the internal stress state within a tissue. For example, a decrease of approximately 1 nm has been associated with enzymatic degradation of cartilage tissue, and a decrease of 0.2 nm [40] was related to significant internal stress *in vivo* on the collagen network in corneal tissue [41].

D-Period measurements have also been reported in the literature to compare different tissue types. For example, AFM has been used to characterise differences in D-Period in the cornea and sclera for both mouse [42] and human [43] tissues. In these studies, the D-Period in the sclera was found to be 3.3 nm and 2.4 nm lower than the cornea for the mouse and human respectively. These differences are thought to be related to differing ECM arrangements in the two types of tissue, for example, related to differences in proteoglycans and glycosaminoglycans (GAGs) surrounding the collagen fibrils.

More recently, a number of studies have shown that collagen D-Period distributions change as a function of disease, as reviewed by Chen et al. [44]. We also observed statistically significant differences in the D-Period distribution in the low and high PWV groups. This motivated us to analyze the data further by splitting the D-Period measurements into three groups hence allowing us to clearly identify the trends in D-Period beyond the expected range (59–70 nm) [30], [31]. We noticed that the most significant difference between the low and high PWV groups was found in the low D-Period range (45–59 nm) and the high D-Period range (70–80 nm), as shown in Fig. 7C and E. Although it is now clear from our study and others [44] that heterogeneity present in the collagen D-Period appears to be important for understanding tissue properties and function, there is still a gap in our understanding about why these changes occur.

To date, there are limited studies on collagen fibril D-Period in vascular tissues. However, a recent study on a mouse model of abdominal aortic aneurysms (AAA) used D-Period measurements from AFM and transmission electron microscopy (TEM) to investigate whether there was a difference in collagen fibril ‘quality’ in AAA tissue relative to controls [30]. AFM revealed much more variability in the AAA tissue and TEM revealed statistically significant differences relative to controls. Hence, the collagen D-Period serves as a good indicator of changes within the tissue’s local environment.

It should be noted that our AFM image analysis routine enabled a large dataset for D-Period to be determined whereas previous studies utilizing AFM have been limited by manual measurements on a small number of images e.g. [43].

### 4.4 Limitations

Our study provides valuable insight into the utility of the IMA for arterial stiffening investigations, however there are a number of limitations which should be addressed. Firstly, although we compare the mechanical properties of the IMA with carotid-femoral PWV we acknowledge that the IMA is not involved in carotid-femoral PWV pathway and our work does not provide a mechanistic link between the two. Secondly, due to the number of different tests that were conducted on the IMA samples in parallel studies it was not possible to compare the AFM data with established, functional biomechanical tests such as wire or pressure myography. Thirdly, the study only focusses on adventitial collagen, whilst other studies on the IMA (including our prior proteomics work) are based on measurements across the entire vessel i.e. intima-adventitia. Future studies would benefit from accompanying data on the medial layer. Fourthly, we were not able to provide any histological quantification of collagen content which would have complemented the AFM work. Fifthly, alterations in other molecular components of the adventitia may be contributing to elastic modulus differences that we found. However, such studies are beyond the scope of the present work. Finally, we were limited by a sample size of seventeen patients. However, we note this is a larger sample size than other relevant adventitial biomechanics studies where samples sizes of six [20], eleven [7] and thirteen [21] have been reported for example.

## 5 Conclusions

The adventitia is an important layer of arteries in terms of both biomechanics and physiology of blood vessels, but in terms of arterial stiffening it has received little attention relative to the medial layer. Our study on adventitial nanomechanics in the IMA compares patients with low and high PWV. We have shown that the IMA is an ideal vessel for nano-scale *in vitro* experimentation because it is readily accessible during coronary artery bypass operations, and appears to reflect changes across the vasculature although it is not an elastic vessel and hence does not contribute to PWV measurements. We found that the nanomechanical properties of the adventitia correlated with clinical assessment of arterial stiffness (via carotid-femoral PWV) and also downregulation of extracellular proteins that have already been shown to be associated with arterial stiffness. Interestingly, these extracellular proteins (SLRPs) are known to be involved in collagen fibrillogenesis [19] and we found that ultrastructural properties of the adventitial collagen fibrils differed in the low and high PWV groups. Our extensive dataset on collagen fibril diameter and D-Period served as a suitable method for detecting alterations in the local adventitial environment. Our findings highlight the important role of the adventitia in arterial stiffening and the suitability of the IMA as a target vessel for biomechanical studies. We suggest that measurements of collagen fibril diameter and D-Period are excellent nano-scale markers for assessing collagen fibril and adventitial ‘quality’ in pathology. Our approach provides a method of characterizing small biopsy samples to predict the development of arterial stiffening. This will be an invaluable approach for future studies developing new therapeutic targets for tackling arterial stiffening.

## References

[1] Laurent S., Cockcroft J., Van Bortel L., Boutouyrie P., Giannattasio C., Hayoz D., Pannier B., Vlachopoulos C., Wilkinson I., Struijker-Boudier H. (2006). Expert consensus document on arterial stiffness: methodological issues and clinical applications. Eur. Heart J..

[2] Meaume S., Benetos A., Henry O.F., Rudnichi A., Safar M.E. (2001). Aortic pulse wave velocity predicts cardiovascular mortality in subjects >70 years of age. Arterioscler. Thromb. Vasc. Biol..

[3] R. Akhtar, B. Derby, Mechanical Properties of Aging Soft Tissues, 2015.

[4] Sawabe M. (2010). Vascular aging: from molecular mechanism to clinical significance. Geriatr. Gerontol. Int..

[5] Akhtar R., Graham H.K., Derby B., Sherratt M.J., Trafford A.W., Chadwick R.S., Gavara N. (2016). Frequency-modulated atomic force microscopy localises viscoelastic remodelling in the ageing sheep aorta. J. Mech. Behav. Biomed. Mater..

[6] Akhtar R. (2014). In vitro characterisation of arterial stiffening: from the macro-to the nano-scale. Artery Res..

[7] Schulze-Bauer C.A., Regitnig P., Holzapfel G.A. (2002). Mechanics of the human femoral adventitia including the high-pressure response. Am. J. Physiol. Heart Circul. Physiol..

[8] Kohn J.C., Lampi M.C., Reinhart-King C.A. (2015). Age-related vascular stiffening: causes and consequences. Front. Genet..

[9] Lu X., Pandit A., Kassab G.S. (2004). Biaxial incremental homeostatic elastic moduli of coronary artery: two-layer model. Am. J. Physiol. Heart Circulat. Physiol..

[10] Grant C.A., Twigg P.C. (2013). Pseudostatic and dynamic nanomechanics of the tunica adventitia in elastic arteries using atomic force microscopy. ACS Nano.

[11] Hu Y., Zhang Z., Torsney E., Afzal A.R., Davison F., Metzler B., Xu Q. (2004). Abundant progenitor cells in the adventitia contribute to atherosclerosis of vein grafts in ApoE-deficient mice. J. Clin. Investig..

[12] El Kasmi K.C., Pugliese S.C., Riddle S.R., Poth J.M., Anderson A.L., Frid M.G., Li M., Pullamsetti S.S., Savai R., Nagel M.A. (2014). Adventitial fibroblasts induce a distinct proinflammatory/profibrotic macrophage phenotype in pulmonary hypertension. J. Immunol..

[13] Majesky M.W., Dong X.R., Hoglund V., Daum G., Mahoney W.M. (2012). The adventitia: a progenitor cell niche for the vessel wall. Cells Tissues Organs.

[14] Young T.J., Monclus M.A., Burnett T.L., Broughton W.R., Ogin S.L., Smith P.A. (2011). The use of the PeakForce TM quantitative nanomechanical mapping AFM-based method for high-resolution Young's modulus measurement of polymers. Meas. Sci. Technol..

[15] Papi M., Paoletti P., Geraghty B., Akhtar R. (2014). Nanoscale characterization of the biomechanical properties of collagen fibrils in the sclera. Appl. Phys. Lett..

[16] Faarvang A.S.A., Rørdam Preil S.A., Nielsen P.S., Beck H.C., Kristensen L.P., Rasmussen L.M. (2016). Smoking is associated with lower amounts of arterial type I collagen and decorin. Atherosclerosis.

[17] Cangemi C., Skov V., Poulsen M.K., Funder J., Twal W.O., Gall M.-A., Hjortdal V., Jespersen M.L., Kruse T.A., Aagard J., Parving H.-H., Knudsen S., Høilund-Carlsen P.-F, Rossing P., Henriksen J.E., Argraves W.S., Rasmussen L.M. (2011). Fibulin-1 is a marker for arterial extracellular matrix alterations in type 2 diabetes. Clin. Chem..

[18] Preil S.A.R., Kristensen L.P., Beck H.C., Jensen P.S., Nielsen P.S., Steiniche T., Bj??rling-Poulsen M., Larsen M.R., Hansen M.L., Rasmussen L.M. (2015). Quantitative proteome analysis reveals increased content of basement membrane proteins in arteries from patients with type 2 diabetes mellitus and lower levels among metformin users. Circ. Cardiovasc. Genet..

[19] Hansen M.L., Beck H.C., Irmukhamedov A., Jensen P.S., Olsen M.H., Rasmussen L.M. (2015). Proteome analysis of human arterial tissue discloses associations between the vascular content of small leucine-rich repeat proteoglycans and pulse wave velocity. Arterioscl. Thrombosis Vasc. Biol..

[20] Hoffman A.H., Teng Z., Zheng J., Wu Z., Woodard P.K., Billiar K.L., Wang L., Tang D. (2017). Stiffness properties of adventitia, media, and full thickness human atherosclerotic carotid arteries in the axial and circumferential directions. J. Biomech. Eng..

[21] Holzapfel G.A., Sommer G., Gasser C.T., Regitnig P. (2005). Determination of layer-specific mechanical properties of human coronary arteries with nonatherosclerotic intimal thickening and related constitutive modeling. Am. J. Physiol. Heart Circulat. Physiol..

[22] C. Reference Values for Arterial Stiffness (2010). Determinants of pulse wave velocity in healthy people and in the presence of cardiovascular risk factors: 'establishing normal and reference values'. Eur Heart J..

[23] Heilbronner R., Barrett S. (2013). Image Analysis in Earth Sciences: Microstructures and Textures of Earth Materials.

[24] Schneider C.A., Rasband W.S., Eliceiri K.W. (2012). NIH Image to ImageJ: 25 years of image analysis. Nat. Methods.

[25] Benjamini Y., Hochberg Y. (1995). Controlling the false discovery rate – a practical and powerful approach to multiple testing. J. R. Stat. Soc. B Met..

[26] Team R.C. (2014). R: A Language and Environment for Statistical Computing.

[27] Borovic M.L., Borovic S., Peric M., Vukovic P., Marinkovic J., Todorovic V., Radak D., Lackovic V. (2010). The internal thoracic artery as a transitional type of artery: a morphological and morphometric study. Histol. Histopathol..

[28] Merrilees M.J., Tiang K.M., Scott L. (1987). Changes in collagen fibril diameters across artery walls including a correlation with glycosaminoglycan content. Connect. Tissue Res..

[29] Parry D.A., Barnes G.R., Craig A.S. (1978). A comparison of the size distribution of collagen fibrils in connective tissues as a function of age and a possible relation between fibril size distribution and mechanical properties. Proc. R. Soc. Lond. B Biol. Sci..

[30] Tonniges J.R., Albert B., Calomeni E., Hans C., Agarwal G. (2016). Ultrastructural imaging of collagen fibrils in mouse model of abdominal aortic aneurysm. Microsc. Microanal..

[31] Fang M., Goldstein E.L., Turner A.S., Les C.M., Orr B.G., Fisher G.J., Welch K.B., Rothman E.D., Banaszak Holl M.M. (2012). Type I collagen D-spacing in fibril bundles of dermis, tendon, and bone: bridging between nano- and micro-level tissue hierarchy. ACS Nano.

[32] Millasseau S.C., Stewart A.D., Patel S.J., Redwood S.R., Chowienczyk P.J. (2005). Evaluation of carotid-femoral pulse wave velocity. Hypertension.

[33] Chung A.W., Booth A.D., Rose C., Thompson C.R., Levin A., Van Breemen C. (2008). Increased matrix metalloproteinase 2 activity in the human internal mammary artery is associated with ageing, hypertension, diabetes and kidney dysfunction. J. Vasc. Res..

[34] Ergul A., Portik-Dobos V., Hutchinson J., Franco J., Anstadt M.P. (2004). Downregulation of vascular matrix metalloproteinase inducer and activator proteins in hypertensive patients. Am. J. Hypertens.

[35] Chen S., Birk D.E. (2013). The regulatory roles of small leucine-rich proteoglycans in extracellular matrix assembly. FEBS J..

[36] Kalamajski S., Oldberg Å. (2010). The role of small leucine-rich proteoglycans in collagen fibrillogenesis. Matrix Biol..

[37] Goh K.L., Holmes D., Lu Y., Purslow P.P., Kadler K., Béchet D., Wess T.J. (2012). Bimodal collagen fibril diameter distributions direct age-related variations in tendon resilience and resistance to rupture. J. Appl. Physiol..

[38] Robinson K.A., Sun M., Barnum C.E., Weiss S.N., Huegel J., Shetye S.S., Lin L., Saez D., Adams S.M., Iozzo R.V., Soslowsky L.J., Birk D.E. (2017). Decorin and biglycan are necessary for maintaining collagen fibril structure, fiber realignment, and mechanical properties of mature tendons. Matrix Biol..

[39] Tzaphlidou M., Berillis P. (2004). Effect of lithium administration on collagen and breaking pressure of the rat thoracic descending aorta. J. Trace Elements Exp. Med..

[40] Inamdar S.R., Knight D.P., Terrill N.J., Karunaratne A., Cacho-Nerin F., Knight M.M., Gupta H.S. (2017). The secret life of collagen: temporal changes in nanoscale fibrillar pre-strain and molecular organization during physiological loading of cartilage. ACS Nano.

[41] Bell J.S., Hayes S., Whitford C., Sanchez-Weatherby J., Shebanova O., Vergari C., Winlove C.P., Terrill N., Sorensen T., Elsheikh A., Meek K.M. (2018). The hierarchical response of human corneal collagen to load. Acta Biomater.

[42] Miyagawa A., Kobayashi M., Fujita Y., Nakamura M., Hirano K., Kobayashi K., Miyake Y. (2000). Surface topology of collagen fibrils associated with proteoglycans in mouse cornea and sclera. Jpn. J. Ophthalmol..

[43] Miyagawa A., Kobayashi M., Fujita Y., Hamdy O., Hirano K., Nakamura M., Miyake Y. (2001). Surface ultrastructure of collagen fibrils and their association with proteoglycans in human cornea and sclera by atomic force microscopy and energy-filtering transmission electron microscopy. Cornea.

[44] Chen J., Ahn T., Colon-Bernal I.D., Kim J., Banaszak Holl M.M. (2017). The relationship of collagen structural and compositional heterogeneity to tissue mechanical properties: a chemical perspective. ACS Nano.

