# Supplementary material for: Nanomechanics and ultrastructure of the internal mammary artery adventitia in patients with low and high pulse wave velocity
Source: Acta Biomater. 2018 Jun;73:437–48. doi: 10.1016/j.actbio.2018.04.036 (PMC5995416; doi:10.1016/j.actbio.2018.04.036)
Supplement: Supplementary data 1 [file mmc1.docx]

**Nanomechanics and ultrastructure of the internal mammary artery adventitia in patients with low and high pulse wave velocity**

Zhuo Chang^1^, Paolo Paoletti^1^, Steve D. Barrett^2^, Ya Hua Chim^1^, Eva Caamaño-Gutiérrez^3^, Maria Lyck Hansen^4^, Hans Christian Beck^4^, Lars Melholt Rasmussen^4^, Riaz Akhtar^1*^

^1^ Department of Mechanical, Materials and Aerospace Engineering, School of Engineering, University of Liverpool, L69 3GH, UK.

^2^ Department of Physics, University of Liverpool, Liverpool, L69 7ZE, UK.

^3^Computational Biology Facility, Institute of Integrative Biology, University of Liverpool, Liverpool, L69 7ZB

^4^Department of Clinical Biochemistry and Pharmacology, Center for Individualized Medicine in Arterial Diseases, Odense University Hospital, University of Southern Denmark.

**Supplementary Material**

Table S1. Summary of the patient numbers and subsequent tests that were conducted on tissue from each patient (n=8 and n=9 for the low and high PWV group). Nanomechanical analysis was conducted on tissue sections for each patient. Of these patients, collagen fibril diameter and D-Period was determined for every patient except for 559 due to unreliable data obtained from the image analysis routine. Hence, there were 16 patients included in the collagen fibril morphology analysis (n=7 and n=9 for the low and high PWV group, respectively). The proteomics data was based on a previous study by Hansen et al. [19]. Hansen et al. collected proteomics data to determine SLRP expression for 12 of the patients included in our study (n= 6 in both the low and high PWV groups).

| Patient No. | Number on PCA | Nanomechanical properties | | Collagen fibril morphology | SLRPs expression | | |
| --- | --- | --- | --- | --- | --- | --- | --- |
| Low PWV |  |  | |  |  | | |
| 522 | 1 | Y | | Y | | Y | |
| 549 | 2 | Y | | Y | | N | |
| 552 | 3 | Y | | Y | | N | |
| 559 | 4 | Y | | N | | Y | |
| 565 | 5 | Y | | Y | | Y | |
| 631 | 6 | Y | | Y | | Y | |
| 656 | 7 | Y | | Y | | Y | |
| 693 | 8 | Y | | Y | | Y | |
| High PWV |  |  | |  | |  | |
| 524 | 9 | Y | | Y | | Y | |
| 534 | 10 | Y | | Y | | Y | |
| 573 | 11 | Y | Y | | | | N |
| 603 | 12 | Y | Y | | | | Y |
| 606 | 13 | Y | Y | | | | Y |
| 620 | 14 | Y | Y | | | | N |
| 627 | 15 | Y | Y | | | | Y |
| 643 | 16 | Y | Y | | | | N |
| 1202 | 17 | Y | Y | | | | Y |


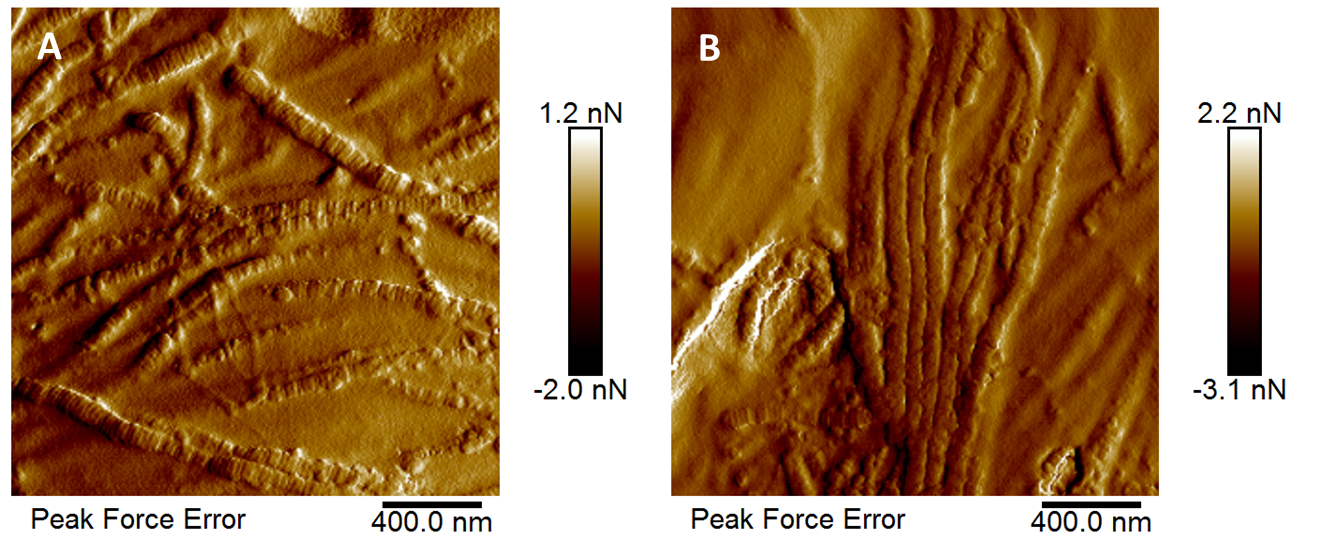


Figure S1 Peak Force Error AFM images (2 µm × 2 µm) of the adventitial layer of patient 559. The images were composed of loosely packed collagen fibrils and hence were unsuitable for the image analysis routine which considered the fibrils as contiguous rectangular objects.


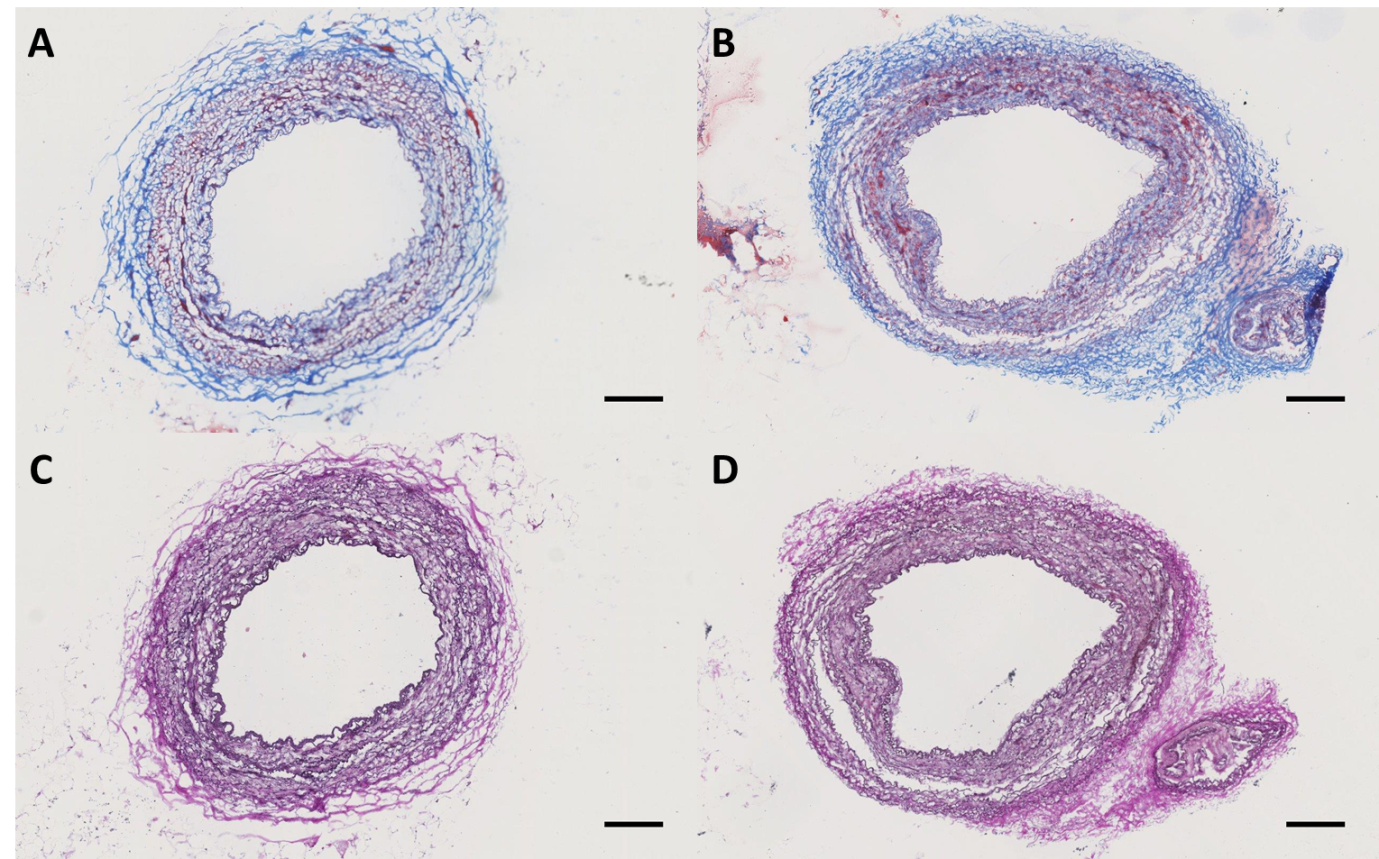


Figure S2 Masson’s staining for collagen (A) Patient 559 and (B) Patient 620. Weigert’s stain for elastin (C) Patient 559 (D) Patient 620. Both of these patients had higher levels of elastin than the others in the study. Scale bar indicated 200 μm length.


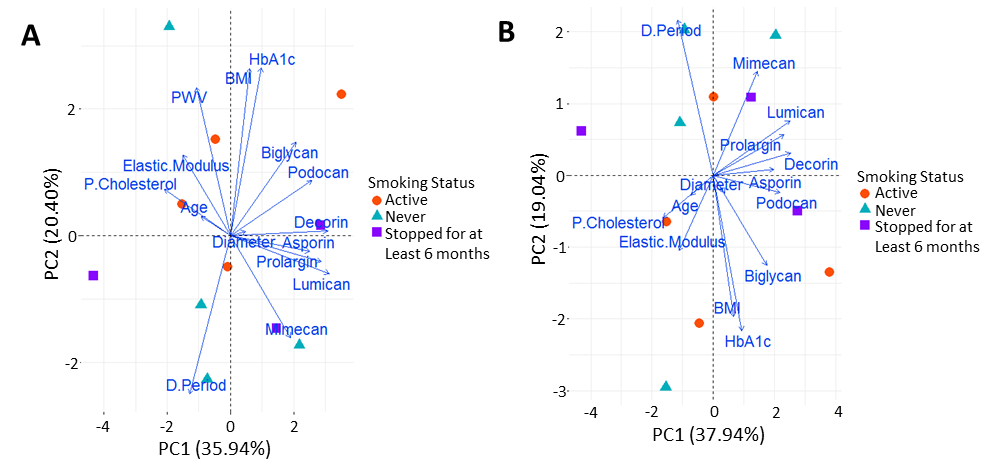


Figure S3 Principal component analysis plot with smoking history overlaid to the plot for each patient; (A) with PWV variable and (B) without PWV variable.
